# Knowledge of HIV/AIDS among married women in Bangladesh: analysis of three consecutive multiple indicator cluster surveys (MICS)

**DOI:** 10.1186/s12981-022-00495-8

**Published:** 2022-12-28

**Authors:** Mohammad Nayeem Hasan, Sumi Tambuly, Kaniz Fatema Trisha, Md. Ashiqul Haque, Muhammad Abdul Baker Chowdhury, Md Jamal Uddin

**Affiliations:** 1 Department of Statistics, Shahjalal University of Science & Technology, Sylhet, Bangladesh; 2 Department of Community Health Sciences, University of Manitoba, Winnipeg, Canada; 3 Department of Neurosurgery, University of Florida College of Medicine, Gainesville, FL USA; 4 Department of General Educational Development (GED), Daffodil International University, Dhaka, Bangladesh

**Keywords:** Multiple Indicator Cluster Survey (MICS), HIV/AIDS, Knowledge, Mass media, Global health, Married women, Women health

## Abstract

Married women have a higher risk of contracting human immunodeficiency virus (HIV) or develop acquired immune deficiency syndrome (AIDS) than men. Knowledge of HIV/AIDS contributes significantly to describing the prevalence and consequences of such virus/disease. The study aimed to investigate the level of HIV/AIDS knowledge and the socio-demographic variables that influence HIV/AIDS knowledge among married women in Bangladesh. We used three waves of Multiple Indicator Cluster Survey (MICS), which included 33,843, 20,727, and 29,724 married women from 2006, 2012, and 2019 MICS. A score was prepared through their interrogation to determine the level of knowledge and logistic regression models were used for analyzing the data. This study found that the prevalence of knowledge level of HIV/AIDS in different questions increased from 55.20% in 2006 to 58.69% in 2019. In our study, respondents having highest education had 4.03 (95% CI 3.50–4.64) times more chance to obtain “High Score” in 2019 MICS which is 5.30 times in 2012 MICS (95% CI 4.41–6.37) and 2.58 times in 2006 MICS (95% CI 2.28–2.93) compared to illiterate married women. Moreover, respondents from urban area were 1.13 times more likely to obtain “High Score” in 2019 MICS which is 1.14 times in 2012 MICS and 1.16 times in 2006 MICS, respectively than the rural married women. This study also found respondent’s age, division, mass media access, and wealth status have played an important role in HIV/AIDS knowledge. Although a significant proportion of women had adequate knowledge of HIV/AIDS, more knowledge is still required to protect against such viruses/diseases. Thus, we advocate for the implementation of educational program in the curriculum, counselling, particularly in rural areas, and mass media access to ensure quality knowledge throughout the country.

**Supplementary Information:**

The online version contains supplementary material available at 10.1186/s12981-022-00495-8.

## Introduction

The Human Immunodeficiency Virus (HIV)/Acquired Immune Deficiency Syndrome (AIDS) is one of the world's most widespread pandemic affecting over 38.4 million individuals were living worldwide and 1.5 million people were newly infected with HIV/AIDS et the end of 2021 [1]. According to World Health Organizations (WHO) factsheets, HIV continues to be a serious global public health problem, having lived 40.1 million [33.6–48.6 million] people to date [2]. Despite of major advancements in the information, diagnostics, treatment and prevention of HIV/AIDS, the number of deaths due to HIV and related complications continues to rise [3, 4]. Globally, 770,000 people have died from HIV related causes and 37.9 million people are living with HIV as of 2018. Among all HIV cases, 24.5 million people living with HIV were under antiretroviral therapy, majority of them were married women aged 15–44 because women experience a lot of hormonal changes, microbial ecology and physiology [5–8].

According to UNAIDS, at the end of 2013, 4.8 million peoples in Asia and the Pacific were HIV-positive, with more than 90% of those cases occurring in China, India, Indonesia, Myanmar, Thailand, and Vietnam. The consequences of HIV-related deaths in Asia decreased by 37% between 2005 and 2013 [9].

In Bangladesh, where the prevalence of AIDS is low, the virus affects 0.1 percent of the general population. However, study shows that new infected cases are increasing steadily. Total 5,586 HIV positive cases had been found in Bangladesh of whom 865 were new cases. The majority of newly diagnosed HIV-positive individuals were centered in the divisions of Dhaka (54%), Chittagong (21%), Khulna (10%), and Sylhet (6%) [9]. The number of HIV-infected homemakers and pregnant mothers has increased in the last five years. Growth in female cases is seen worldwide as feminization and early sign of epidemics [10].

Women in Bangladesh are considered to be exposed with a high risk of HIV due to lack of opportunity for general and health education because of gender inequality and male dominance [11]. According to numerous studies, men in Bangladesh have a better degree of knowledge than women [12]. In most cases, women share a large percentage of the disease's consequences due to their favorable socio-economic status, limited access of sexually and reproductive health care [13, 14] and that is why women are at higher risk for HIV infection and transmission. It is also true that women in Bangladesh are contaminated with myths and rumors about HIV/AIDS which increase the number of HIV infections or transmissions [15]. The factors responsible for expanding the infections of HIV in Bangladesh are mainly poverty, medical facilities, education, lack of sufficient screening practices, and unprotected sexual practices [12]. Especially in the southern part of Bangladesh, with the influx of forcibly displaced Myanmar nationals or the Rohingya people in 2017, the risk of HIV infections increased due to increased sharing of syringes, needles, unprotected sexual practices and most importantly lack of knowledge [16].

The HIV infection is incurable and it has become a manageable chronic health condition, allowing people living with HIV to lead long and healthy lives. This is due to increased access to effective HIV prevention, diagnosis, treatment, and care, including for opportunistic infections [2]. Many studies have been conducted to know the level of knowledge about HIV/AIDS among the married women in Bangladesh using both primary and secondary data. Similar findings were found in several studies which can relate with our study [11, 16]. Since there is no cure still invented and there is now a high risk of spreading in Bangladesh, higher level of knowledge is needed among the people. In this situation, further research can show the present view of knowledge level among the married women. In Bangladesh, however, empirical research on knowledge of HIV with the comparison of different survey data is lacking. Therefore, in this study, we aimed to explore the level of HIV knowledge among the married women in Bangladesh, using three waves of Multiple Indicator Cluster Survey (MICS) dataset. We also intend to find out the factors associated with knowledge of HIV.

## Materials and methods

### Data source and study variables

We used three waves of Multiple Indicator Cluster Survey (MICS) conducted in 2006, 2012, and 2019. MICS is a large, multidimensional nationally representative household survey conducted by the UNICEF. This survey uses standardized questionnaires to provide the information and key indicators on the situation of children. Most often, they concentrate on mother and child health interventions, early childhood development, child nutrition status, and reproductive health. MICS also collects a uniform set of socio-economic characteristics of individuals and households [17, 18]. Datasets were open access in the public domain [19].

### Sampling design and sample size

The MICS survey is a two-stage cluster sampling procedure, randomly selecting women with reproductive age. MICS-2006 is based on a sample of 78,260 women interviewed with a response rate of 92.5%, MICS-2012 is based on a sample of 59,599 women interviewed with a response rate of 98.5% and 2019 MICS is based on a sample of 68,709 interviewed with a response rate of 99.4%. Based on the expected sizes of the enumeration areas from the most recent population census, primary sampling units (PSUs), which were used to determine the census enumeration areas, were chosen from each sample domain using a systematic PPS (probability proportional to size) sampling approach. MICS provides a comprehensive picture of children and women’s health in the seven administrative divisions (Barisal, Chittagong, Dhaka, Khulna, Rajshahi, Rangpur, and Sylhet) of Bangladesh. Districts were identified as the primary sample strata for sample selection at two stages [17, 18].

### Outcome

Respondents who have ever heard of AIDS were asked 9 HIV related questions in 2012 and 2019 MICS survey and 10 questions were asked in 2006 MICS survey (Table 1). Where for each of the question, 1 was assigned for the correct answer and 0 was coded for the ‘wrong’ or ‘don’t know’. Based on the summation of the scores, knowledge score was prepared which was a count variable. Using the prepared count variable a dichotomous variable was prepared based on median, which we took as outcome variable in this study. For clear, in 2006 MICS survey, median was taken as 8 whereas medians were taken as 5 and 6 for 2012 and 2019 MICS survey. It had a potential range of 0 to 10, with higher scores indicating more knowledge about HIV. Finally, scores greater than or equal to the median were assigned to the ‘High Score’ category and the remaining scores were assigned to the ‘Low Score’ [20].

**Table 1:** Comparison of correct response rate between three consecutive MICS survey

Questions about HIV related knowledge	Correct response					
	2006		2012		2019	
	Yes N (%)	No (%)	Yes N (%)	No (%)	Yes N (%)	No (%)
% of people aware that transmission can be avoided by:						
Having a one faithful and uninfected sex partner	25,340 (75.34)	8503 (24.66)	11,640 (58.13)	9087 (41.87)	9783 (32.61)	19,941 (67.39)
Always using a condom	22,226 (65.89)	11,617 (34.11)	10,617 (52.31)	10,110 (47.69)	18,277 (62.42)	11,447 (37.58)
Even someone who appears healthy could be sick	25,850 (75.85)	7993 (24.15)	11,668 (56.15)	9059 (43.85)	17,212 (58.30)	12,512 (41.70)
% of married women who know that HIV cannot be spread by:						
Food sharing is not a method of HIV transmission	16,926 (49.43)	16,917 (50.57)	10,409 (52.14)	10,318 (47.86)	24,258 (82.46)	5466 (17.54)
It is impossible for mosquito bites to spread HIV	14,667 (43.60)	19,176 (56.40)	8638 (42.99)	12,091 (57.01)	13,609 (53.02)	16,115 (46.98)
HIV cannot be spread via paranormal means	24,223 (71.97)	9620 (28.03)	14,440 (70.80)	6287 (29.20)	14,903 (50.84)	14,821 (49.16)
Sharing needles is one method of HIV transmission	30,994 (91.50)	2849 (8.50)	–	–		
% of women who are aware that HIV can be passed from mother to child:						
HIV transmitted while pregnant	30,373 (89.71)	3470 (10.29)	15,078 (71.92)	5649 (28.08)	21,827 (72.57)	7897 (27.43)
HIV transmitted during childbirth	26,055 (76.73)	7788 (23.27)	9546 (42.26)	11,181 (57.74)	17,298 (43.05)	12,426 (56.95)
Breast milk is a source of HIV transmission	30,182 (89.04)	3661 (10.96)	14,100 (65.83)	6627 (34.17)	22,344 (75.11)	7380 (24.89)

### Covariates

A set of covariates such as ten years age group (15–24, 25–34, 35–44, 45 +), residency type, respondent’s education, religion, wealth index, household’ education level, access to mass media and religion were used for this study. There were three categories for the mother's educational background: no education, primary and secondary completion or higher (completing at least grade 10). High economic class was reclassified as having an asset value of 20% or more, middle economic class having an asset value of 40% or more, and low economic class having an asset value of 40% or less [21]. Access to mass media variable was generated by respondent’s condition to reach to at least one of three mediums television, newspaper or radio.

### Statistical analysis

By modifying the survey's sampling weight, the descriptive statistics of each of the chosen covariates and the distribution of HIV score categories were displayed. The association between the HIV score category and other socio-demographic factors was also determined using weighted percentages and Pearson's Chi-Squad test. We first used logistic regression models because our result is a binary variable. Then, considering every relevant covariate together with the HIV score category variable, we built univariate models. Covariates were only allowed to be included in multivariable models if their p-value was less than 0.05. The best model was chosen using stepwise procedures, and it comprised all relevant covariates as well as some important outcome-related variables. The Area under the Receiver Operating Characteristic (AUROC), the indicators of sensitivity, specificity, Calibration belt plot and Hosmer–Lemeshow goodness of fit test to evaluate the accuracy of best model. The higher ROC areas indicated a better performance of the models. In ROC curve, lower P-value conclude that the model actually does discriminate between two categories and area under curve is higher than the 0.50 [22]. Calibration and Hasmer–Lemesho goodness of the fit test measures how good the model-estimated probabilities is similar with the observed outcomes and it is usually evaluated through a goodness-of-fit test. Calibration belt plot and Hosmer–Lemeshow goodness of fit test with P-value greater than 0.05 suggest the ability of the model to correctly classify observations into outcome categories [23, 24].

To consider complex survey design, we used the Svyset command in Stata (Statacorp LP, College Station, Texas). Svyset commands help us to use design elements such as PSU, strata, clusters and sample weights [25].

### Ethics approval

Our study was based entirely on an analysis of public domain health survey datasets from the MICS 2006, 2012, and 2019 that are publicly available online and have been removed of any personally identifying information. Both UNICEF and the Bangladesh Bureau of Statistics (BBS) examined and approved the MICS methods. Informed consent was obtained from participants while interviewing them. Because this study involved the analysis with secondary data thus, it did not require the ethical approval of the respective institution.

## Results

**Fig. 1:**
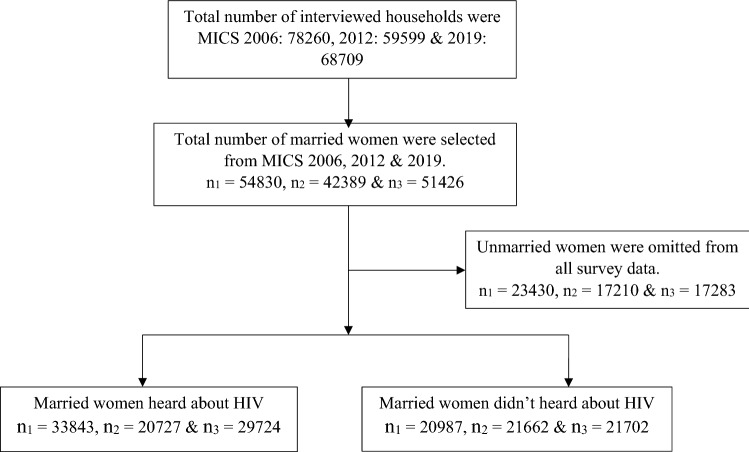
Schematic diagram of the analytic study sample

**Fig. 2:**
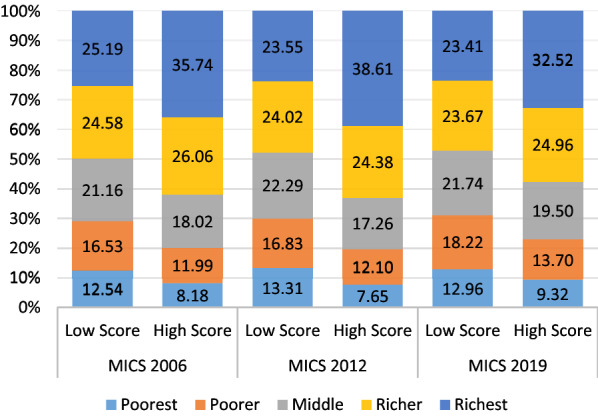
Distribution of HIV score category of married women by wealth index

**Fig. 3:**
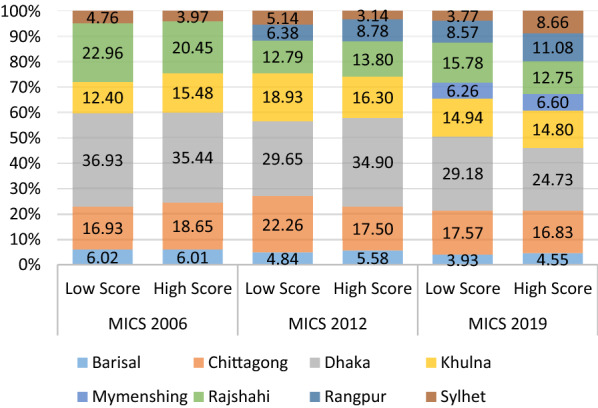
Distribution of HIV score category of married women by region

This study included 33,843 in 2006, 20,727 in 2012, and 29,724 in 2019 married women who heard about HIV (Fig. 1). The prevalence of achieved high score in knowledge questions increased from 55.20% in 2006 to 58.69% in 2019. According to wealth index, 38.61% respondents categorized by richest wealth index scored ‘high score’ in 2012 MICS survey against 2006 MICS survey (35.74%) though it shows a decreasing rate in 2019 (32.52%) (Fig. 2). In Sylhet division, following 2019 MICS survey, 8.66% respondents scored high score regarding HIV knowledge whereas in 2006 and 2012 MICS, these were 3.97% and 3.14% respectively. However, individuals from the Dhaka division had the lowest percentage of high scores (24.73%) in 2019, down from 34.90% and 35.44% in the MICS of 2012 and 2006 survey (Fig. 3).

Table 1 shows the comparison of correct response rate over a thirteen-year period (2006 to 2019). Percentage of correct response to the questions about HIV related knowledge have fallen all over the period for most of the questions except the questions about using of condom every time which shows a drop down in 2012 against 2006 (65.89% to 52.31%) but shows a risen loop in 2019 against 2012 (52.31% to 62.42%). Table 1 also shows changes in the percentage of people who know HIV transmission is not possible by sharing food. The overall status of the percentage of knowing that HIV cannot be transmitted by food is 49.43% according to 2006 MICS survey whereas it is increased 52.14% in 2012 MICS survey and this percentage increased incredibly in 2019 showing 82.46%.

Table 2 represents the status of respondents affecting the levels of HIV score category by their socio-demographic characteristics by using chi square test showing the p-values. HIV score among the young people is higher than the other age groups people. Percentages of high score in 15–24 age group are 55.84%, 66.29% and 60.16% according to 2006, 2012 and 2019 MICS survey, respectively. In addition, an increasing rate of high score is clearly understood in the age group 45 and above from 2006 to 2019 which are 48.17%, 58.58% respectively in 2006 and 2012. Though it started decreasing in 2019 (52.13%).

**Table 2:** HIV/AIDS knowledge score status at different levels of covariates

Covariates	MICS 2006			MICS 2012			MICS 2019		
	Low Score n (%)	High Score n (%)	P-value	Low Score n (%)	High Score n (%)	P-value	Low Score n (%)	High Score n (%)	P-value
Total	15,160 (44.80)	18,683 (55.20)	–	7277 (35.07)	13,450 (64.93)	–	12,113 (41.31)	17,611 (58.69)	–
Ten years age group									
15–24	5218 (44.16)	6540 (55.84)	0.000	2027 (33.71)	4131 (66.29)	0.000	3148 (39.84)	4971 (60.16)	< 0.001
25–34	5225 (43.60)	6831 (56.40)		2882 (32.62)	5694 (67.38)		4775 (40.01)	7352 (59.99)	
35–44	3545 (45.75)	4209 (54.25)		1836 (39.60)	2896 (60.40)		3220 (43.26)	4276 (56.74)	
45 and above	1172 (51.83)	1103 (48.17)		532 (41.42)	729 (58.58)		970 (47.87)	1012 (52.13)	
Division									
Barisal	1517 (44.88)	1825 (55.12)	0.000	597 (31.88)	1276 (68.12)	0.000	849 (37.85)	1223 (62.15)	< 0.001
Chittagong	2451 (42.45)	3440 (57.55)		1263 (40.73)	1913 (59.27)		1935 (42.36)	2931 (57.64)	
Dhaka	4547 (45.85)	5224 (54.15)		1715 (31.46)	3543 (68.54)		2904 (45.37)	3376 (54.63)	
Khulna	2379 (39.43)	3621 (60.57)		1789 (38.55)	3209 (61.45)		2413 (41.53)	3476 (58.47)	
Mymenshing	–	–		–	–		615 (40.01)	926 (59.99)	
Rajshahi	3315 (47.70)	3565 (52.30)		868 (33.37)	1660 (66.63)		1804 (46.55)	1987 (53.45)	
Rangpur	–	–		553 (28.20)	1317 (71.8)		1045 (35.25)	2131 (64.75)	
Sylhet	951 (49.35)	1008 (50.65)		492 (46.91)	532 (53.09)		548 (23.44)	1561 (76.56)	
Type of place of residence									
Rural	9360 (48.04)	10,120 (51.96)	0.000	1335 (27.95)	3321 (72.05)	0.000	2587 (36.58)	4586 (63.42)	< 0.001
Urban	5513 (39.81)	8109 (60.19)		5942 (38.05)	10,129 (61.95)		9526 (43.07)	13,025 (56.93)	
Tribal	287 (45.75)	454 (54.25)		–	–		–	–	
Women Highest educational level									
No education	4208 (57.36)	3167 (42.64)	0.000	1358 (55.74)	1169 (44.26)	0.000	1236 (57.52)	904 (42.48)	< 0.001
Primary incomplete	2535 (51.90)	2356 (48.10)		1163 (52.39)	1169 (47.61)		–	–	
Primary complete	2272 (47.50)	2520 (52.50)		1198 (44.92)	1464 (55.08)		2911 (54.19)	2524 (45.81)	
Secondary incomplete	4507 (40.08)	6710 (59.92)		2853 (32.54)	5977 (67.46)		6515 (42.36)	9300 (57.64)	
Secondary completed or Higher	1637 (29.71)	3929 (70.29)		705 (15.93)	3671 (84.07)		1451 (22.87)	4883 (77.13)	
Religion									
Islam	13,264 (45.22)	15,939 (54.78)	0.003	6450 (35.35)	11,836 (64.75)	0.322	10,939 (41.61)	15,617 (58.39)	0.0229
Others	1896 (41.64)	2743 (58.36)		827 (33.62)	1614 (66.38)		1174 (38.36)	1994 (61.64)	
Accessibility to mass media									
Do not have mass media access	–	–	–	3842 (45.25)	9055 (54.75)	0.000	3890 (45.90)	4648 (54.10)	< 0.001
Have mass media access	–	–		3435 (29.90)	4395 (70.10)		8223 (39.68)	12,963 (60.32)	
Husband/partner’s education level									
No education	5463 (52.12)	5033 (47.88)	0.000	2450 (35.45)	4423 (64.55)	0.682	3293 (48.37)	3638 (51.63)	< 0.001
Primary	3756 (47.56)	4177 (52.44)		1786 (34.41)	3429 (65.59)		3544 (46.03)	4306 (53.97)	
Secondary or Higher	5856 (38.42)	9383 (61.58)		2640 (35.05)	4909 (64.95)		3779 (40.23)	5790 (59.77)	
Non-standard curriculum	36 (45.76)	47 (54.24)		–	–		1492 (28.25)	3873 (71.75)	
Wealth Index									
Poorest	1910 (55.46)	1470 (44.54)	0.000	1185 (48.47)	1263 (51.53)	0.000	1828 (49.47)	1923 (50.53)	< 0.001
Poorer	2547 (52.83)	2332 (47.17)		1363 (42.89)	1930 (57.11)		2431 (48.35)	2673 (51.65)	
Middle	3240 (48.82)	3497 (51.18)		1660 (41.09)	2581 (58.91)		2733 (43.96)	3666 (56.04)	
Richer	3830 (43.39)	5065 (56.61)		1691 (34.73)	3471 (65.27)		2787 (40.03)	4468 (59.97)	
Richest	3633 (36.41)	6319 (63.59)		1378 (24.78)	4205 (75.22)		2334 (33.62)	4881 (66.38)	
Household’s Head Sex									
Male	14,384 (44.92)	17,662 (55.08)	0.327	5976 (34.65)	11,279 (65.35)	0.077	11,061 (41.35)	16,037 (58.65)	0.704
Female	776 (43.39)	1021 (56.61)		905 (37.36)	1487 (62.64)		1052 (40.91)	1574 (59.09)	

*Scores greater than or equals to the median were assigned to ‘High score’ category and the rest scores were treated as to fall in the category, ‘Low score’

Respondents living in rural area (63.42%) scored high score in 2019 compared to urban people’s scoring status (56.93%) of 2019 MICS survey. High scoring status of rural area were 51.96% and 72.05% according to 2006 and 2012 MICS survey. Over thirteen-year time, household’s education level shows a significant change in score of HIV knowledge. In 2019 MICS survey, 77.13% respondents having secondary or higher education level scored ‘high’ which was 70.29% in 2006 MICS survey. Married women who have access of mass media shows the HIV knowledge status as 60.32% respondents scored high score in 2019 against 2012 MICS survey (70.10%).

Table 3 portrays the outcome of binary logistic regression analysis of HIV/AIDS related knowledge, counting 95% CI for odds ratio. In brief, respondents aged 25–34 years were 1.11 (95% CI 1.04–1.19) times more likely to get ‘High Score’ in 2019 which is comparatively smaller than 2012 MICS’s “High Score” [1.20 (95% CI 1.09–1.32)] and greater than 2006 MICS’s 1.06 (95% CI 0.99–1.13) compared to 15–19 years married women. According to MICS 2019, HIV knowledge in married women from Sylhet division were 2.42 (95% CI 2.08–2.83) times more likely to get “High Score” which is lower in 2012 MICS 0.51 (95% CI 0.42–0.61), 2006 MICS (OR = 0.83, 95% CI 0.71–0.98), respectively compared to Barisal division. As expected, respondents from urban area were 1.13 (95% CI 1.04–1.22) times more chance to obtain “High Score” compare to rural area in 2019 MICS which is smaller in 2012 MICS 1.14 (95% CI 1.01–1.29) and 2006 MICS 1.16 (95% CI 1.06–1.26), respectively than the rural married women. Respondents who have mass media access were 1.13 (95% CI 1.05–1.21) times more chance to obtain “High Score” in 2019 MICS which is smaller than 2012 MICS 1.36 (95% CI: 1.24–1.48) compared to married women who have not mass media access. Respondents having highest education had 4.03 (95% CI: 3.50–4.64) times more chance to obtain “High Score” in 2019 MICS which is 5.30 times in 2012 MICS (95% CI: 4.41–6.37) and 2.58 times in 2006 MICS (95% CI 2.28–2.93) compared to illiterate married women.

**Table 3:** Factors associated with the HIV/AIDS knowledge score of married women, MICS 2006, MICS 2012 and 2019

Covariates	MICS 2006		MICS 2012		MICS 2019	
	OR (95% CI)	P-value	OR (95% CI)	P-value	OR (95% CI)	P-value
Ten years age group						
15–24	1		1		1	
25–34	1.06 (0.99–1.13)	0.081	1.20 (1.09–1.32)	0.000	1.11 (1.04–1.19)	0.003
35–44	1.02 (0.95–1.10)	0.588	1.03 (0.92–1.17)	0.810	1.06 (0.98–1.15)	0.138
45 and above	0.81 (0.72–0.91)	0.001	0.96 (0.80–1.16)	0.756	0.94 (0.84–1.07)	0.399
Division						
Barisal	1		1		1	
Chittagong	1.03 (0.89–1.19)	0.692	0.61 (0.51–0.72)	0.000	0.78 (0.69–0.89)	< 0.001
Dhaka	0.97 (0.84–1.11)	0.644	0.89 (0.77–1.04)	0.141	0.67 (0.59–0.76)	< 0.001
Khulna	1.31 (1.14–1.49)	0.000	0.84 (0.73–0.97)	0.033	0.87 (0.77–0.98)	0.025
Mymenshing	–	–	–	–	1.01 (0.86–1.18)	0.947
Rajshahi	0.91 (0.79–1.04)	0.178	0.94 (0.81–1.11)	0.531	0.71 (0.63–0.81)	< 0.001
Rangpur	–	–	1.14 (0.96–1.36)	0.196	1.17 (1.02–1.34)	< 0.001
Sylhet	0.83 (0.71–0.98)	0.024	0.51 (0.42–0.61)	0.000	2.42 (2.08–2.83)	0.022
Type of place of residence						
Rural	1		1		1	
Urban	1.16 (1.06–1.26)	0.001	1.14 (1.01–1.29)	0.058	1.13 (1.04–1.22)	0.003
Tribal	1.02 (0.76–1.37)	0.901	–	–	–	–
Women Highest educational level						
No education	1		1		1	
Primary incomplete	1.20 (1.09–1.32)	0.001	1.12 (0.97–1.29)	0.084	–	–
Primary complete	1.42 (1.30–1.55)	0.001	1.48 (1.28–1.71)	0.000	1.12 (0.99–1.26)	0.059
Secondary incomplete	1.83 (1.68–2.00)	0.001	2.44 (2.13–2.79)	0.000	1.77 (1.58–1.99)	< 0.001
Secondary completed or Higher	2.58 (2.28–2.92)	0.001	5.30 (4.41–6.37)	0.000	4.03 (3.50–4.64)	< 0.001
Religion						
Islam	1		1		1	
Others	1.07 (0.97–1.18)	0.199	1.02 (0.90–1.18)	0.743	1.01 (0.89–1.13)	0.904
Accessibility to mass media						
Do not have mass media access	–	–	1		1	
Have mass media access	–	–	1.36 (1.24–1.48)	0.000	1.13 (1.05–1.21)	< 0.001
Husband/partner’s education level						
No education	1		1		1	
Primary	1.02 (0.95–1.10)	0.595	1.04 (0.94–1.16)	0.421	1.01 (0.93–1.09)	0.889
Secondary or Higher	1.06 (0.98–1.14)	0.156	1.03 (0.94–1.14)	0.516	1.08 (0.99–1.17)	0.071
Non-standard curriculum	1.18 (0.70–2.00)	0.526	–		1.21 (1.08–1.35)	0.001
Wealth Index						
Poorest	1		1		1	
Poorer	1.01 (0.92–1.12)	0.793	1.07 (0.94–1.22)	0.295	1.01 (0.91–1.12)	0.902
Middle	1.12 (1.01–1.24)	0.038	1.01 (0.87–1.15)	0.949	1.10 (0.99–1.22)	0.086
Richer	1.26 (1.14–1.40)	0.000	1.13 (0.97–1.31)	0.112	1.19 (1.06–1.32)	0.002
Richest	1.32 (1.16–1.49)	0.000	1.26 (1.04–1.52)	0.018	1.21 (1.06–1.37)	0.004

Our model fitting criteria the AUC of receiver operating characteristic curve (ROC) was found to be 0.624 (Asymptotic p-value: 0.000 and 95% CI 0.619–0.66), 0.677 (Asymptotic p-value: 0.000 and 95% CI 0.669–0.685), and 0.657 (Asymptotic p-value: 0.000 and 95% CI 0.651–0.663) indicating the final selected model for MICS-2006, MICS-2012, and MICS-2019 respectively showed higher area under curve than 0.50. The output of the Calibration belt plot reports that the p-value of MICS-2006, MICS-2012 and MICS-2019 were 0.845, 0.680, and < 0.001 which suggest that the hypothesis of good calibration is not rejected in MICS-2006 and MICS-2012 model but the calibration is not good for MICS-2019. According to Hosmer–Lemeshow chi-squared and p-value, the MICS-2006 model fitted best following MICS-2012 and MICS-2019. Additional file 1: Fig. S1 also illustrate classification plots, which intersect sensitivity and specificity in a probability cutoff value. The intersect point is equivalent to classification accuracy of the model. The classification accuracy of MICS-2006, MICS-2012 and MICS-2019 were 59.92%, 66.97%, and 63.03%, respectively (Additional file 1: Fig. S2 and Table S1).

## Discussion

We investigated the level of knowledge about HIV/AIDS among married women in Bangladesh. This study identified some socio-demographic and background determinants of HIV knowledge such as age, residence type, level of education, wealth index, and so on. We observed that a large proportion of women (58.69%) had a "High Score" about HIV-related knowledge, according to the latest available MICS data. The figure was lower than that of a comparative study conducted in 13 countries of sub-Saharan Africa; in which DHS data in each country was used; shows higher level of HIV/AIDS knowledge among the women as those countries experienced HIV pandemic earlier [11, 12]. Although those who have HIV are typically most contagious in the first few months after becoming infected, many don't become aware of their condition until much later. People may suffer no symptoms in the initial weeks following infection or flu-like symptoms such as fever, headache, rash, or sore throat [2].

Married women had a high level of knowledge about HIV transmission and prevention methods. However, there were a few misconceptions, the most notable of which was the idea that wearing condoms can prevent HIV transmission. In each study, more than 50% of respondents were aware that condom use can prevent HIV transmission. Knowledge of condom using as a transmission reduction for HIV was higher in a study among international students in China [26]. Blood, breast milk, semen, and vaginal secretions are just a few of the bodily fluids from infected people that can spread HIV. During pregnancy and delivery, a mother's HIV infection might pass to her kid. Normal daily interactions like kissing, hugging, shaking hands, or sharing of personal items, food, or water do not cause an infection in a person. Knowledge was increased in huge in Bangladesh regarding the transmission way by sharing foods, 49.3% to 82.46% in 2006 to 2019 survey, response correctly in this question. A study conducted in rural Kenya, most of the respondents knew HIV cannot spread by sharing food [27].

In this study, mass media accessibility was significantly associated with HIV/AIDS knowledge. Many countries used mass communication through mass media to raise knowledge of HIV/AIDS [11]. Thus, proper use of mass media plays a significant role in increasing knowledge of HIV/AIDS. In our study, household wealth status was significantly associated with levels of HIV knowledge. Poorest were less knowledge to be in the “High Score” group of HIV than for those who were in the richest group.

Findings of our study showed people of age group 25–34 years tend to have high HIV knowledge compared to the people of other age groups, reflecting similarities with previous study [28]. Since people of this age group generally get in touch with social platform and other ways of mass media, which support them to increase the HIV knowledge among them. One Serbia based study revealed that 53% women of Serbian women aged 15–49 are likely to have immense knowledge about HIV/AIDS [29]. A mixed model analysis of BDHS pooled data describes that women of different age group have different level of HIV/AIDS knowledge whereas women aged 20–29 years are more aware of HIV than young married women (15–19 years) [28].

Level of education was also an important influencing factor among the determinants since this study revealed people of having secondary completed or higher education tend to have more HIV knowledge than other, which is consistent with other previous studies [13, 30]. Previous studies have identified that women's education has a significant impact and that current research increases the likelihood of having higher HIV knowledge. The level of educational attainment had a significant impact on HIV/AIDS knowledge. With each year of schooling increases, the level of knowledge increased significantly. This findings is in line with earlier research [13].

Our study also identified that married women who have access to mass media were likely to have higher knowledge about HIV. Earlier publications showed the similar result about the influence of mass media accessibility regarding HIV knowledge among the married women [31]. Similarly, richest respondents showed having comparatively higher knowledge regarding HIV than the other categories of wealth-indexed people.

In our study, the level of household wealth was significantly associated with HIV knowledge. The "High Score" group of HIV knowledge was less likely to include the poorest than it was to include the richest. The idea that essential necessities like healthy living, education, and healthcare services that create the environment for effective health communication and knowledge acquisition are not directly impacted by economic well-being. [13]. Because the people of this category have easy access to mass media and tend of having higher education which help them to know more about HIV. Previous publication also showed same result for this category of people [12, 31].

We also noticed variations in knowledge levels regarding HIV observed among the different people in different administrative divisions in Bangladesh. People living in Sylhet districts had higher HIV knowledge in 2019 MICS compared to other division. This clearly indicates the unequal coverage of knowledge building programs regarding HIV, so implementation of such programs in all divisions need to be prioritized. However, in an earlier study, people of Barisal division were found to be more aware regarding HIV knowledge [31].

## Strengths and limitations

To the best of our knowledge, this is the first study to compare three waves of MICS survey data in the context of HIV/AIDS knowledge among Bangladeshi married women. We used a sufficiently large nationally representative dataset that reflects Bangladesh's whole population. We also took into account a wide range of factors that influence the public's knowledge of the issue. We also looked at model-fitting criteria, which were mostly absent in the literature.

Despite this, there were certain limits to our research. Because we used secondary data, we had no control over variable selection, data quality, or measurement indications. Furthermore, the study was performed three years ago; in that period, the level of knowledge among respondents may have shifted.

## Conclusion and recommendations

This study revealed the levels of knowledge regarding among the married women both from rural and urban area of Bangladesh associated with some influencing factors of HIV knowledge such as mass media, levels of education, wealth index, and type of living place, age groups and divisions. According to this study, women with less education and access to the media are less knowledgeable about HIV; as a result, initiatives for education that specifically target people with less education and limited media access are required. In addition, to transmit a more accurate and nuanced understanding of HIV transmission and acquisition, education subjects should match specific subtopics. Although several married women were found to be acknowledged regarding HIV, more initiatives should be taken to implement HIV knowledge related programs in all divisions, especially in Rajshahi and Chittagong. Age specific initiative should be taken for making knowledgeable to HIV infections, mostly in 45 years or above married women. Government, Non-governmental organizations, program organizers and policy makers should work together to implement the knowledge raising strategies and facilitate more educational interventions among the married women of primary or lower educational status and for rural women. Thus, a strategic plan and proper implementation should be implied to mitigate the looming threat of an HIV/AIDS epidemic.

## Supplementary Information

**Additional file 1:** **Figure S1.** Sensitivity analysis of fitted final multivariable logistic regression model. **Figure S2.** Calibration belt plot. **Table S1.** Hosmer-Lemeshow Test, Area under ROC Curve, and Calibration test and classification accuracy for final logistic regression model.

## Data Availability

All data presented here in the manuscript is freely available at dhsprogram.com.
