# Supplementary material for: Knowledge of HIV/AIDS among married women in Bangladesh: analysis of three consecutive multiple indicator cluster surveys (MICS)
Source: AIDS Res Ther. 2022 Dec 28;19:68. doi: 10.1186/s12981-022-00495-8 (PMC9795636; doi:10.1186/s12981-022-00495-8)
Supplement: Supplementary file 1 — Additional file 1: Figure S1. Sensitivity analysis of fitted final multivariable logistic regression model. Figure S2. Calibration belt plot. Table S1. Hosmer-Lemeshow Test, Area under ROC Curve, and Calibration test and classification accuracy for final logistic regression model. [file 12981_2022_495_MOESM1_ESM.docx]

**Additional Files**

| 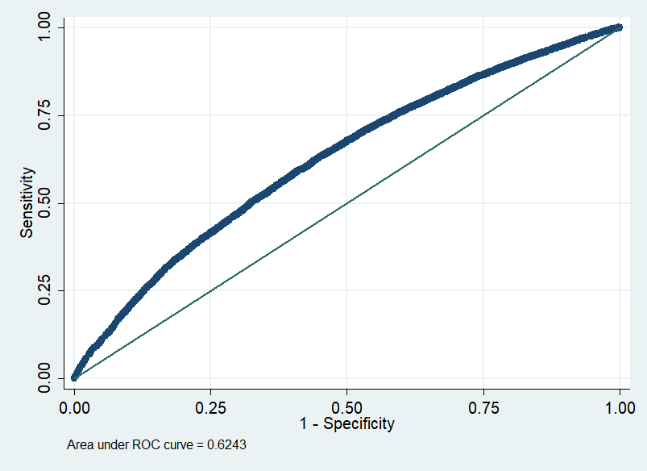 | 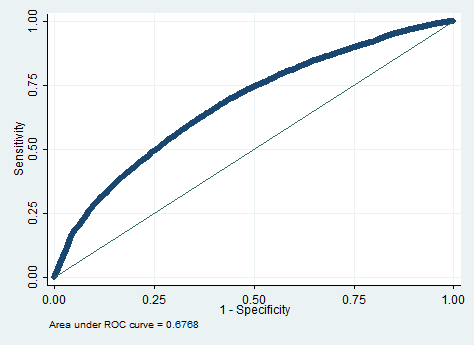 | 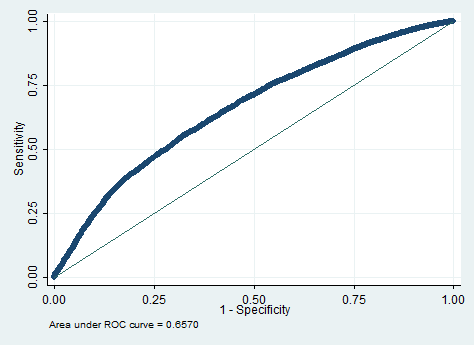 |
| --- | --- | --- |
| 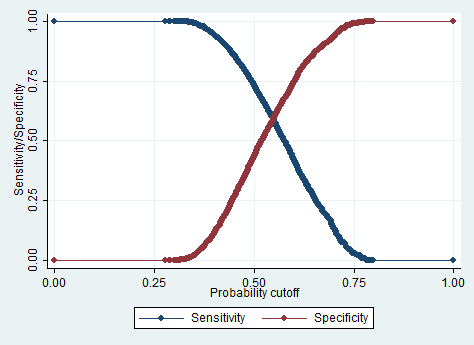 | 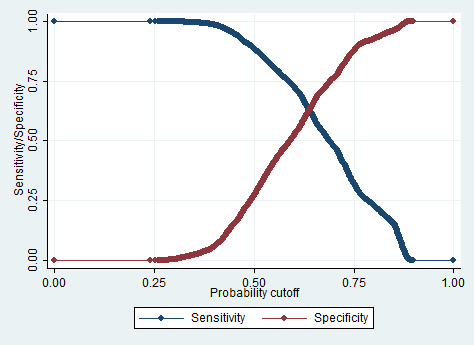 | 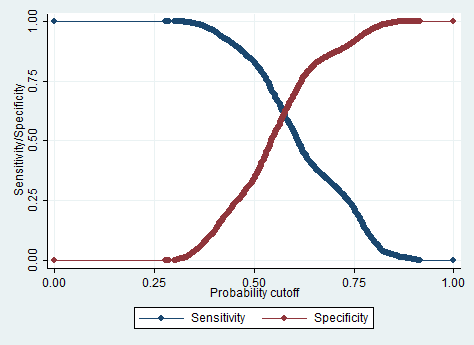 |
| **Figure S1. Sensitivity analysis of fitted final multivariable logistic regression model** | | |
| 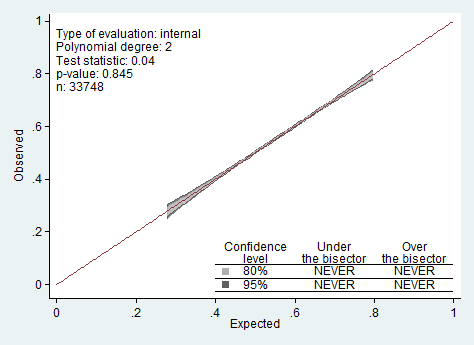 | 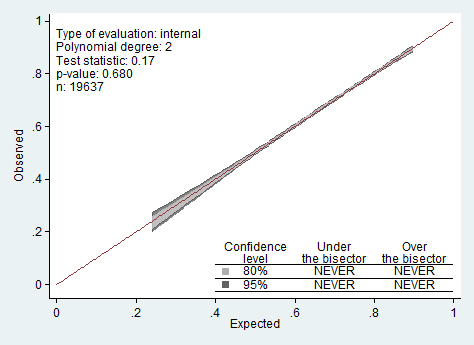 | 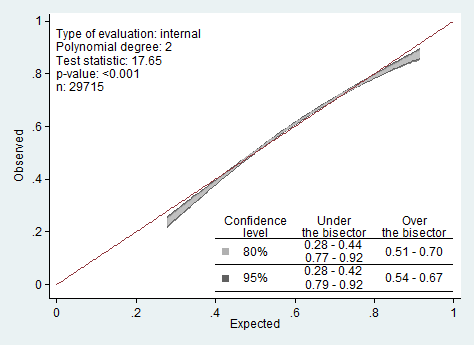 |
| MICS-2006 | MICS-2012 | MICS-2019 |
| **Figure S2: Calibration belt plot** | | |

**Table S1: Hosmer-Lemeshow Test, Area under ROC Curve, and Calibration test and classification accuracy for final logistic regression model.**

| Survey Year | Hosmer-Lemeshow Test | | Area under ROC Curve | | Calibration test | | Correctly classified |
| --- | --- | --- | --- | --- | --- | --- | --- |
|  | Chi- square | P-value | AUC | P-value | Test-statistic | P-value |  |
| MICS 2006 | 4479.83 | 0.0492 | 0.6243 | <0.001 | 0.04 | 0.845 | 59.92% |
| MICS 2012 | 5498.68 | 0.0038 | 0.6768 | <0.001 | 0.17 | 0.680 | 66.97% |
| MICS 2019 | 6335.91 | 0.0003 | 0.6570 | <0.001 | 17.65 | <0.001 | 63.06% |
